# Analysis of naturally occurring mutations in the human uptake transporter NaCT important for bone and brain development and energy metabolism

**DOI:** 10.1038/s41598-018-29547-8

**Published:** 2018-07-27

**Authors:** Stefan Selch, Anja Chafai, Heinrich Sticht, Andreas L. Birkenfeld, Martin F. Fromm, Jörg König

**Affiliations:** 1 Institute of Experimental and Clinical Pharmacology and Toxicology, Friedrich-Alexander-Universität Erlangen-Nürnberg, Fahrstrasse 17, 91054 Erlangen, Germany; 2 Division of Bioinformatics, Institute of Biochemistry, Friedrich-Alexander-Universität Erlangen-Nürnberg, Fahrstrasse 17, 91054 Erlangen, Germany; 3 Department and Outpatient Department of Medicine III, Carl Gustav Carus University Hospital Dresden, Fetscherstrasse 74, 01307 Dresden, Germany; 4 Paul Langerhans Institute of the Helmholtz Center Munich at the University Hospital and Faculty of Medicine, TU Dresden, Dresden, Germany; 5 German Center for Diabetes Research (DZD e.v.), Neuherberg, Germany

## Abstract

The human uptake transporter NaCT is important for human brain development, brain function and energy metabolism and mediates the uptake of citrate and other intermediates of the tricarboxylic acid cycle from blood into neurons and hepatocytes. Mutations in the *SLC13A5* gene encoding NaCT are associated with epileptic encephalopathy. To gain more insights into the transport mechanisms we analyzed the functional consequences of mutations in the *SLC13A5* gene on NaCT-mediated transport function. Using HEK293 cells expressing wild-type and eight mutated NaCT proteins, we investigated the mRNA and protein amount as well as the protein localization of all NaCT variants. Furthermore, the impact on NaCT-mediated citrate uptake was measured. In addition, a structural model of the transport pore was generated to rationalize the consequences of the mutations on a structural basis. We demonstrated that all proteins were synthesized with an identical molecular weight as the wild-type transporter but several mutations (NaCTp.G219R, −p.G219E, −p.T227M, −p.L420P and −p.L488P) lead to a complete loss of NaCT-mediated citrate transport. This loss of transport activity can be explained on the basis of the developed structural model. This model may help in the further elucidation of the transport mechanism of this important uptake transporter.

## Introduction

Transport proteins belonging to the superfamily of SLC (solute carriers) transporters are important for the uptake of endogenous substances and xenobiotics from the extracellular space into cells. Currently, the SLC superfamily consists of 65 transporter families (SLC1–SLC65) with more than 400 different transport proteins^1^. The *SLC13* gene family of sodium-coupled di- and tricarboxylate/sulfate transporter consists of five transporters (gene symbols *SLC13A1*–*SLC13A5*, transport proteins NaSi1, NaDC2, NaDC3, NaSUT1 and NaCT, respectively) and with orthologues found in prokaryotes and eukaryotes^2^. A member of the SLC13 family, the human uptake transporter NaCT (sodium-coupled citrate transporter; gene symbol *SLC13A5*; also known as mINDY – mammalian INDY) is expressed in the brain and the basolateral membrane of hepatocytes^3^. NaCT is the human orthologue of the Drosophila Indy transporter^4^ and mediates the uptake of citrate and other intermediates of the tricarboxylic acid (TCA) cycle^5^ from blood into neurons and hepatocytes. In *Drosophila melanogaster* and *Caenorhabditis elegans*, reduced expression of this transporter promotes longevity due to caloric restriction^6–8^ and long-lived flies showed an increase in mitochondrial number, a lower expression of insulin-like proteins and reduced body fat stores^9^. In mammals, NaCT has a broad substrate spectrum and shows an inward electrogenic sodium-coupled substrate cotransport with citrate and succinate as important endogenous substrates with high plasma concentrations^5^. Studies with NaCT [INDY (−/−)]-knock out mice demonstrated that deletion of this transporter mimics aspects of dietary restriction and protects these mice against insulin resistance and adiposity^10^. Furthermore, the absence of this transporter led to defective tooth and bone development^11^.

Interestingly, in addition to its role in bone development and energy metabolism, NaCT seems to be important for the development and the function of the human brain because mutations in the *SLC13A5* gene are associated with epileptic encephalopathy [EE^12^]. EE refers to a genetically and clinically heterogeneous group of severe disorders characterized by psychomotor delay, abnormal electro-encephalograms and seizures. Thevenon *et al*.^12^ screened two families and additional unrelated individuals with early-onset EE and detected three mutations (NaCTp.G219R, −p.T227M and −p.L488P) in the *SLC13A5* gene possibly affecting key residues for sodium binding. Recently, these results were confirmed and it has been demonstrated that several other mutations as well as NaCTp.G219R and −p.T227M result in a loss of citrate transport^13,14^ and are associated with neonatal epilepsy, developmental delay and teeth hypoplasia. The mutations NaCTp.G219R and −p.T227M and four novel mutations were examined also in COS-7 cells demonstrating the same effect of NaCTp.G219R and −p.T227M on citrate transport^14^. Interestingly, the phenotype may vary between individuals affected by mutations in the *SLC13A5* gene as shown in a report describing the NaCTp.G219R and the novel −p.Y82C mutants^15^. In this report, the authors described two siblings presented with developmental delay and intractable seizures. Both mutations were found in these siblings but only one individual had focal cortical dysplasia demonstrating the heterogeneity in disease expression even when the genotype is identical.

In 2012, the first structure and proposed transport mechanism of a bacterial sodium-dependent dicarboxylate transporter has been elucidated^16^. Crystallization of the NaCT homologue from *Vibrio cholerae* (vcINDY) showed that this protein functions as a dimer, which has the shape of the letter “M” when viewed from within the membrane plane with one citrate molecule and one sodium molecule bound to each of the monomers^16^. The vcINDY transporter shares 26–33% sequence identity with the human SLC13 family members and catalyzes the sodium-driven uptake of succinate.

To gain more insights into the functional consequences of mutations in the *SLC13A5* gene encoding the human uptake transporter NaCT, we first screened several databases and identified four mutations in the *SLC13A5* gene leading to amino acid substitutions in the NaCT protein (NaCTp.G219E, −p.D243N, −p.L420P and −p.L485R). In addition, we added the three mutations (NaCTp.G219R, −p.T227M and −p.L488P) associated with EE^12^ into this analysis. Furthermore, one mutation known to increase NaCT-mediated citrate transport^4^ was included as positive control (NaCTp.F500L). In total, we investigated the functional consequences of eight mutations (Table 1) in the *SLC13A5* gene leading to amino acid substitutions in the NaCT transporter on protein expression, protein localization and citrate transport. Based on the known structure of the bacterial homologue, we then generated a model of the human NaCT transporter and performed a structural analysis to rationalize the observed effects on the transport of the model substrate citrate.

**Table 1:** Summary of investigated mutations.

Mutation	dbSNP rs# cluster ID	Protein Position	OMIM^®^ *608305	Grantham Score	PolyPhen2 Prediction
c.655G > A	rs144332569	p.G219R	0.0001	125	probably damaging
c.656G > A	rs150024888	p.G219E	None	98	probably damaging
c.680C > T	rs587777577	p.T227M	0.0002	81	probably damaging
c.727G > A	rs142262032	p.D243N	None	23	benign
c.1259T > C	rs150738356	p.L420P	None	98	probably damaging
c.1454T > G	rs148049520	p.L485R	None	102	probably damaging
c.1463T > C	rs587777578	p.L488P	0.0003	98	probably damaging
c.1498T > C	---------------	p.F500L	----------	-----------	benign

## Results

### Analysis of genetic variants in the *SLC13A5* gene

**Figure 1:**
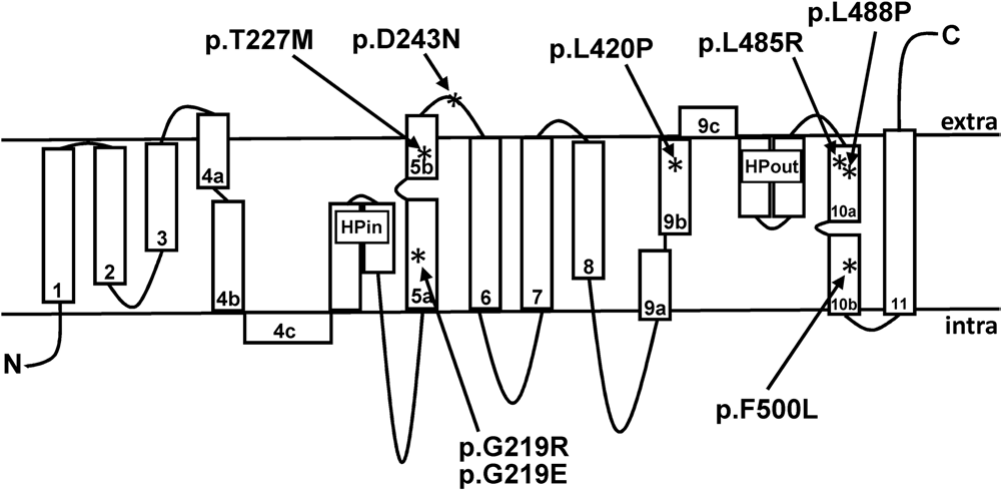
Transmembrane model of human NaCT with the localization of the respective mutant amino acids investigated in this study.

We identified several mutations in the *SLC13A5* gene encoding human NaCT by database search (Table 1) and analyzed their localization in the predicted structure of the transporter (Fig. 1). The first single-nucleotide polymorphisms (SNPs) identified were rs150024888, rs142262032, rs150738356 and rs148049520, leading to the amino acid substitutions NaCTp.G219E, −p.D243N, −p.L420P and −p.L485R (Table 1), respectively. Furthermore, the mutation c.1498T > C (no rs-number) was included into this analysis as a proof of principle, because it is known that the respective amino acid exchange NaCTp.F500L results in a gain-of-function variant leading to a 2- to 3-fold stimulation of citrate uptake into NaCT-transfected cells^4^. Additional variants included into this study originate from the publication by Thevenon and colleagues^12^. They identified three mutations in the *SLC13A5* gene [NaCTp.G219R (rs144332569), NaCTp.T227M (rs587777577) and NaCTp.L488P (rs587777578)] associated with autosomal epileptic encephalopathy. Because no functional characterizations of these mutations have been performed by the time of these experiments, they were included into the set of mutations analyzed in this study. A summary of all investigated mutations together with the *in silico* predicted functional consequences is shown in Table 1.

### Analysis of transiently-transfected HEK293 cells expressing different NaCT mutant proteins

**Figure 2:**
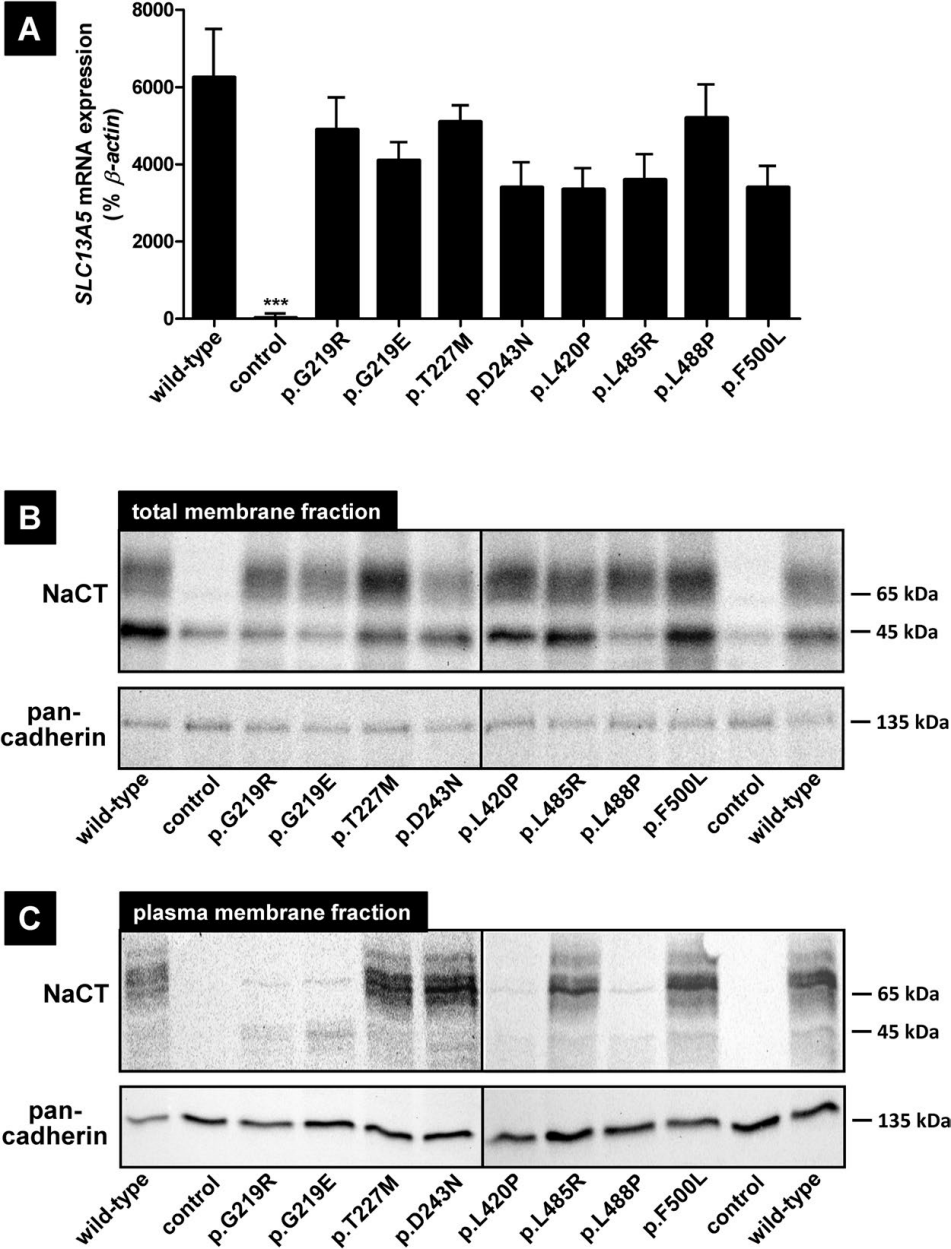
Characterization of transfected HEK293 cells expressing wild-type and mutated NaCT proteins. Characterization of *SLC13A5* mRNA expression (**A**) and of the NaCT protein amount in total membrane fractions (**B**) and in plasma membrane fractions (**C**) in transiently-transfected HEK293 cells. (**A**) Parental HEK293 cells were transiently-transfected with the expression plasmids encoding the wild-type NaCT transporter (wild-type) or with expression plasmids encoding variants of the NaCT protein. Parental HEK293 cells transfected with the empty expression vector served as control (control). All expression values are given in percent ± SD of the respective *ß-actin* mRNA expression measured under the same experimental conditions. (**B**) Immunoblot analysis of total membrane fractions of transiently-transfected HEK293 cells expressing different variants of the NaCT protein. Membrane isolation has been verified by staining the membrane protein pan-cadherin. Presented are the merged pictures of two immunoblots conducted under identical conditions. (**C**) Immunoblot analysis of plasma membrane fractions of transiently-transfected HEK293 cells expressing different variants of the NaCT protein. Membrane isolation has been verified by staining the membrane protein pan-cadherin. Presented are the merged pictures of two immunoblots conducted under identical conditions. The original immunoblots detecting NaCT in the different transient transfections are shown in the supplement (Supplement Figs 1 and 2).

**Figure 3:**
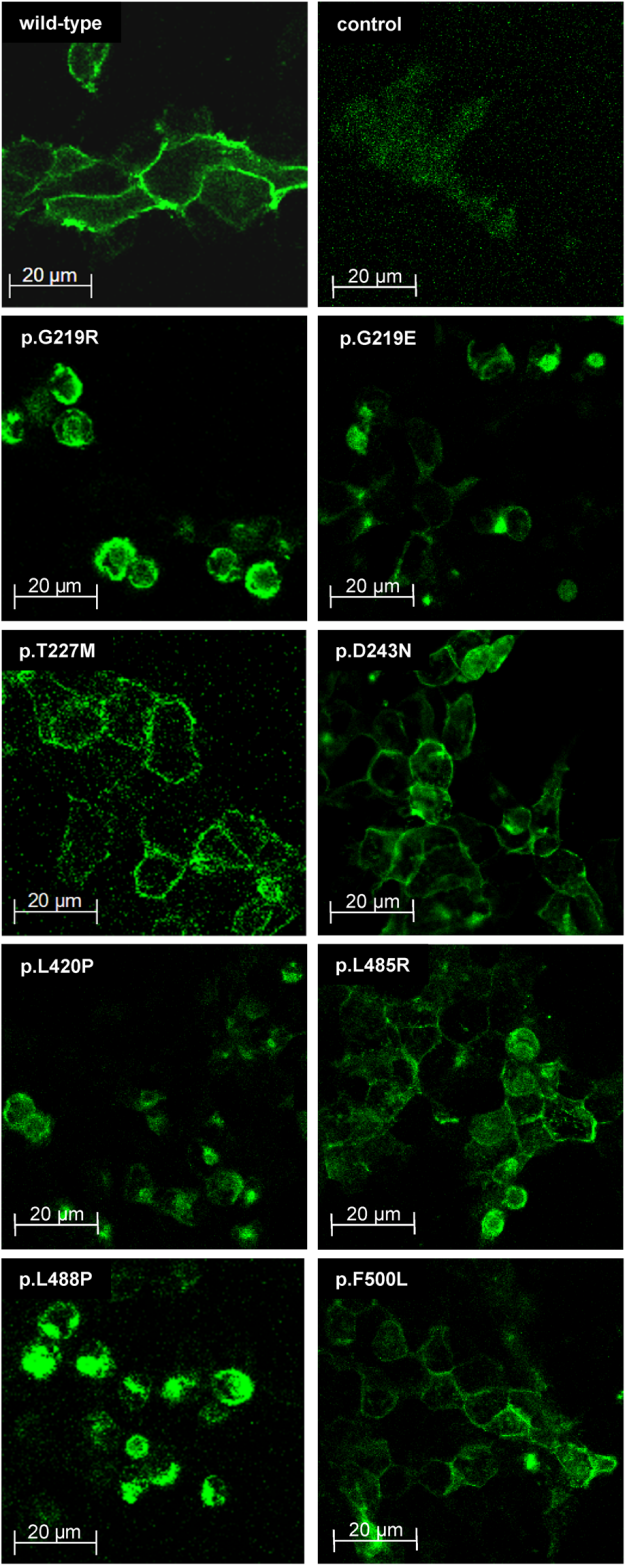
Localization of wild-type and mutated NaCT proteins. Immunofluorescence analysis of NaCT protein localization in transiently-transfected HEK293 cells expressing wild-type or variant NaCT proteins. Empty vector-transfected HEK293 cells served as control.

To compare the functional consequences of the investigated mutations on mRNA expression, protein amount and protein localization, quantitative RT-PCR, immunoblot analyses with total membrane fractions and plasma membrane fractions and confocal laser scanning microscopy were performed. Quantitative RT-PCR (Fig. 2A) demonstrated that *SLC13A5* mRNA expression could be detected in all NaCT transfectants without significant differences, whereas *SLC13A5* mRNA could not be detected in vector-transfected HEK293 cells. The immunoblot analyses (Fig. 2B,C) using the total membrane fraction (Fig. 2B) showed that NaCT protein is detectable in all total membrane fractions except in membrane fractions of vector-transfected control cells suggesting that all mutations have no effect on protein synthesis. Furthermore, all mutated NaCT proteins have the same band pattern as the wild-type NaCT protein. The protein localization analyses (Fig. 3) demonstrated that the mutations NaCTp.T227M, −p.D243N, −p.L485R and −p.F500L showed a localization directly comparable to the localization of the wild-type NaCT protein, whereas no membrane staining could be detected in control cells. Interestingly, mutations NaCTp.G219R, −p.G219E, −p.L420P and −p.L488P showed a slightly altered staining pattern with some NaCT protein retained intracellularly. This intracellular retention could be confirmed by analyzing the plasma membrane fraction (Fig. 2C). This analysis demonstrated that only in the mutants with a localization comparable to the localization of the wild-type protein (NaCTp.T227M, −p.D243N, −p.L485R and −p.F500L) NaCT protein could be detected in comparable amounts in the plasma membrane fraction whereas for all other mutants only a small amount of the protein was found in this fraction (Fig. 2C) suggesting that these mutations also affect protein localization.

### Citrate transport by NaCT mutants

**Figure 4:**
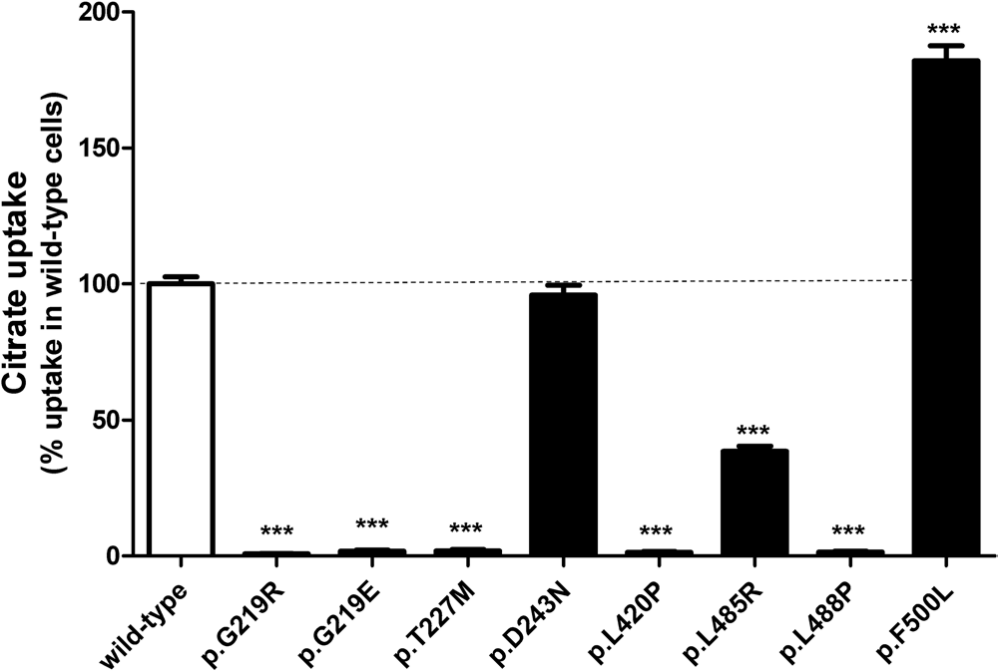
Citrate uptake mediated by wild-type and mutated NaCT proteins. Uptake of citrate (1 µM) into transiently-transfected HEK293 cells. Parental HEK293 cells were transiently-transfected and citrate uptake was measured under established experimental conditions. Cells were transfected with expression plasmids containing cDNAs encoding the wild-type NaCT protein (wild-type) or different mutant NaCT proteins. Citrate net uptake mediated by the wild-type NaCT protein was set to 100% and all net uptake values are given in relation to this uptake ± SD. ***P < 0.001

The effect of the different mutations on NaCT-mediated citrate transport was studied under established transfection and uptake conditions. Using these standardized conditions an uptake ratio of around 70-fold could be detected by comparing the uptake into wild-type transfected HEK cells with empty vector transfected HEK cells serving as control. As expected, the gain-of-function mutant NaCTp.F500L shows an increase in citrate transport of 82% (Fig. 4). A detailed kinetic analysis shown in Table 2 demonstrated that this increase in transport was due to a lower K_m_ value resulting in a higher affinity of this mutant to citrate compared to the wild-type NaCT protein (Table 2). Furthermore, as predicted by *in silico* analysis (Table 1) the mutant −p.D243N has no effect on citrate transport, whereas all other mutants lead to considerably reduced or nearly abolished citrate uptake. Interestingly, the three mutants associated with autosomal epileptic encephalopathy (NaCTp.G219R, −p.T227M and −p.L488P) as well as the mutants −p.G219E and −p.L420P [with high Grantham scores^17^ and predicted to be probably damaging (Table 1)] showed no citrate uptake compared to the wild-type NaCT protein with uptake values comparable to the uptake values in control-transfected cells. The reduced uptake observed for mutant NaCTp.L485R was due to a higher K_m_ value of this mutant for citrate (Table 2) compared to the K_m_ value of the wild-type protein.

**Table 2:** Kinetic parameters of mutants NaCTp.L485R and NaCTp.F500L.

	K_m_ [µM]	V_max_ [pmol × mg protein^−1^ × min^−1^]
NaCT-wild-type	1 439 ± 270.8	13 910 ± 903.2
NaCTp.L485R	4 611 ± 1 849	17 333 ± 3 664
NaCTp.F500L	655.4 ± 370.0	9 335 ± 1 375

### Structural modelling of wild-type and mutant NaCT proteins

**Figure 5:**
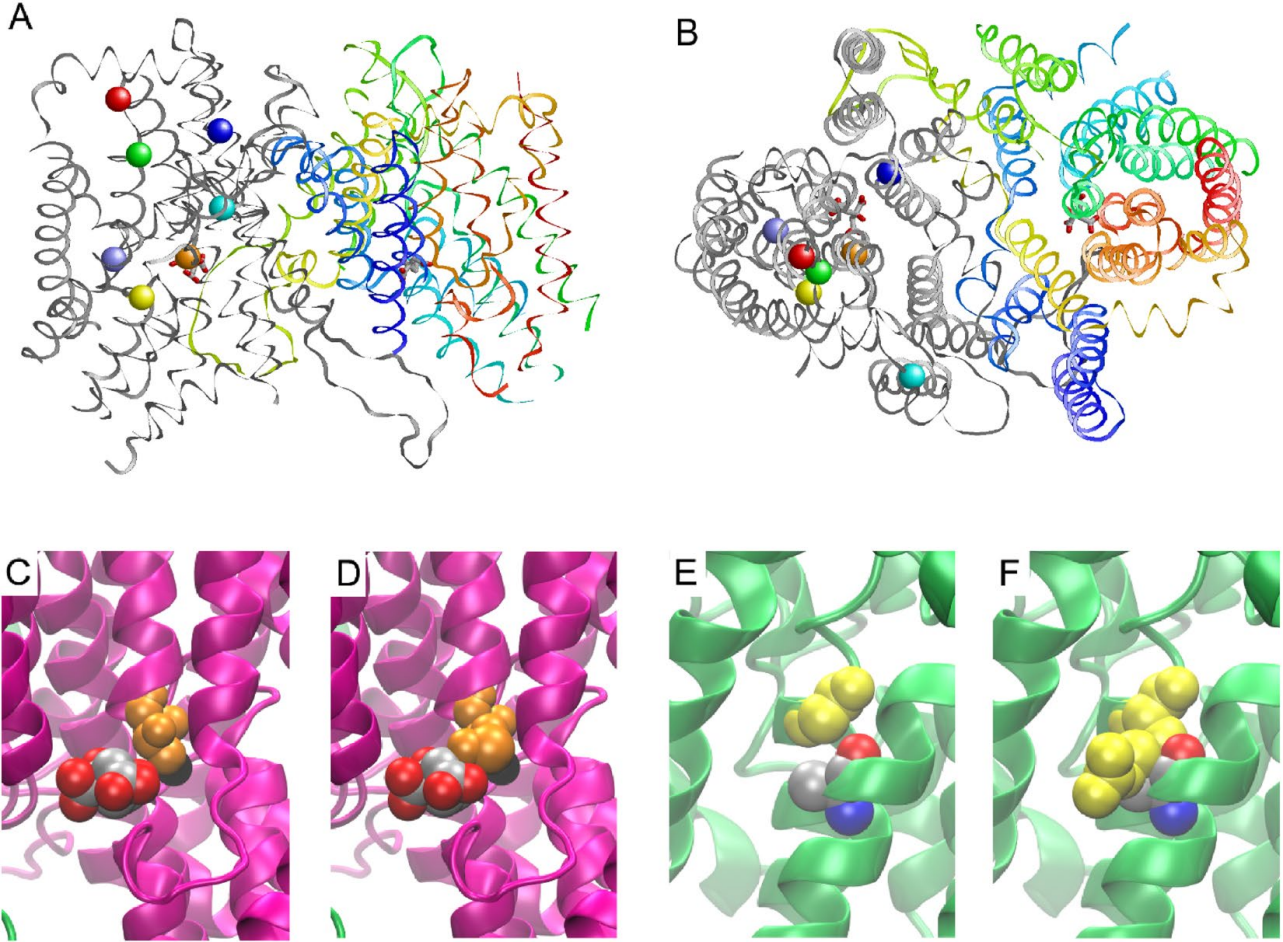
Molecular model of the human NaCT protein and localization of amino acids investigated in this study. (**A**) Molecular model of human NaCT indicating sites of investigated mutations. One of the subunits is shown as grey ribbon and the other subunit is shown in color gradient ranging from blue (N-terminus) to red (C-terminus). In both subunits, a citrate molecule is shown in stick presentation (atom-type coloring). The grey subunit additionally shows the position of the residues investigated by side-directed mutagenesis. These residues are shown in space-filled presentation using the following color code: G219 (yellow), T227 (orange), D243 (cyan), L420 (dark blue), L485 (red), L488 (green) and F500 (light blue). (**B**) Same presentation as in (**A**) but rotated by 90° around the horizontal axis. This corresponds to a view on the NaCT protein from the extracellular site. (**C**,**D**) Effect of the T227M mutation on the NaCT structure. In the wild-type protein, T227 (orange) is close to the substrate citrate (atom-type coloring). (**D**) Due to its longer sidechain M227 (orange) would form clashes with a citrate, which will no longer allow the transport of this substrate. (**E**,**F**) Effect of the G219R mutation on the NaCT structure. In the wild-type protein G219 (yellow) interacts with A133 (atom-type coloring) of an adjacent helix. (**F**) Due to its longer sidechain R219 (yellow) would form clashes with A133 thereby disrupting the NaCT structure.

In order to understand the effects of the mutations on a structural level, a three-dimensional model of the transport pore (the protein region built by the three-dimensional arrangement of the predicted transmembrane helices including residues F11-W116, R123-M159, K202-Y276, F314-G518, and K521-R549) of the NaCT protein with highlighted positions of the investigated amino acids was generated (Fig. 5). The model reveals that the residues G219, T227, L420, L485 and L488 are located within the predicted transport pore, whereas D243 is located distant from the pore and therefore, not influencing transport properties. The sidechain of F500 is oriented away from the pore and forms tight contacts with an adjacent helix. These contacts cannot be formed in the p.F500L mutant, which might lead to an overall enhanced flexibility of the NaCT structure. The residues L420 and L488 are located within helical structures and replacement by the helix breaker proline will disrupt the helical structure resulting in a reduction of transport activity as demonstrated. The effect of the p.T227M mutant is shown in detail in Fig. 5C,D. In the wild-type NaCT protein, T227 is located in immediate vicinity to the citrate molecule (Fig. 5C). In the p.T227M mutant, the sterically more extended methionine sidechain causes steric clashes with the citrate substrate (Fig. 5D) resulting in a pore too small to enable citrate transport. The effect of the G219 mutants is shown in Fig. 5E,F. The molecular modelling reveals that G219 is located at a sterically demanding position within a helix that tightly packs against the residue A113 within a second helix (Fig. 5E). Replacement by glutamate (p.G219E) or arginine [p.G219R (Fig. 5F)] leads to severe clashes with A133 of the second helix, which are predicted to disrupt the structure of the NaCT protein maybe thereby also affecting the membrane localization of the protein.

## Discussion

The aim of this study was to investigate the functional consequences of genetic variations in the *SLC13A5* gene encoding the human uptake transporter NaCT (also known as mINDY) on the transport of the model substrate citrate. NaCT plays an important role in human tooth and bone development, energy metabolism and is crucial for the development and function of the human brain, because mutations in the *SLC13A5* gene are associated with epileptic encephalopathy and developmental delay^12,13^.

Therefore, it is of special interest investigating the functional consequences of mutations and polymorphisms in the *SLC13A5* gene encoding human NaCT. Genetic variations leading to amino acid substitutions were identified by database search. In addition, mutations associated with autosomal recessive encephalopathy^12^ were included into this analysis, three of them (NaCTp.G219R, −p.T227M and −p.L488P) analyzed by *in* *vitro* studies^13,14^. In total, eight mutations leading to amino acid substitutions including one known gain-of-function mutation (p.F500L) were analyzed by transient transfections and uptake studies using citrate as prototypic substrate for this uptake transporter. In addition, consequences of mutations on mRNA expression, protein amount and protein localization were investigated. Immunoblot analyses demonstrated that the molecular weights of all mutated NaCT proteins are comparable to the molecular weight of the wild-type NaCT protein (Fig. 2B) showing for all NaCT proteins the same band pattern. Interestingly, for the mutation NaCTp.G219R, this is in contrast to published data investigating this mutation^13^. For their analyses, Hardies and coworkers used V5-labelled NaCT proteins and an antibody directed against the V5 epitope. Whereas other mutated NaCT proteins could be detected with comparable patterns as the wild-type NaCT protein, the V5-labelled NaCTp.G219R protein was detected as a single protein band with a low molecular weight^13^. In our study, we used untagged NaCT proteins and analyzed total membrane fractions (Fig. 2B) of transfected HEK293 cells demonstrating that all mutated NaCT proteins showed a band pattern comparable to the band pattern of the wild-type NaCT protein, whereas in the cytosolic fraction, no NaCT protein could be detected (data not shown). Our immunoblot data were confirmed in a publication by Klotz and coworkers^14^ were the authors also investigated several mutations in the NaCT transporter including the NaCTp.G219R mutation. In their immunoblot analysis, all mutations showed a band pattern comparable to the wild-type NaCT protein suggesting that the altered staining pattern observed by Hardies *et al*. may be due to the used V5 epitope. The localization of the different NaCT proteins was studied by confocal laser scanning microscopy. This analysis demonstrated that the mutations NaCTp.T227M, −p.D243N, −p.L485R and −p.F500L showed a localization comparable to the localization of the wild-type protein, whereas the staining pattern for the mutations NaCTp.G219R, −p.G219E, −p.L420P and −p.L488P was slightly different with protein being retained intracellularly (Fig. 3). This intracellular retention was confirmed by analyzing the plasma membrane fraction (Fig. 2C) of the wild-type and of all mutant proteins. For the mutants NaCTp.G219R, −p.G219E, −p.L420P and −p.L485P only a small amount of the synthesized protein could be detected in the plasma membrane fraction suggesting that the mutations also affect membrane insertion of the mutated NaCT protein.

Standardized uptake assays using the prototypic NaCT substrate citrate demonstrated that the wild-type protein transports citrate with high uptake values (around 70-fold compared to the uptake into vector-transfected cells), whereas citrate transport mediated by the mutants −p.G219R, −p.G219E, −p.T227M, −p.L420P and −p.L488P was totally abolished (uptake values comparable to uptake values into control-transfected cells) and strongly reduced for the mutant NaCTp.L485R. On one hand, affected transport for the mutants –p.G219R, −p.G219E, −p.L420P and −p.L488P could be due to the altered localization with only a small amount of the protein being inserted into the plasma membrane. However, because wild-type NaCT has an uptake ratio for citrate of around 70-fold (comparing uptake into HEK-NaCT cells with the uptake into control-transfected HEK cells) at least a small protein amount located in the plasma membrane should be reflected in measurable uptake values. Because this is not the case, it is likely that beside the effect of the mutation on membrane localization also the transport function of the mutated NaCT protein is altered. Interestingly, for the mutation NaCTp.T227M, showing no uptake and for the mutation −p.L485R showing strongly reduced uptake, comparable amounts of protein could be detected in the plasma membrane fraction demonstrating that these mutations only affect NaCT transport function. For NaCTp.L485R this reduction was due to an altered K_m_ value resulting in a lower affinity of this mutant to citrate compared to the affinity of the wild-type NaCT protein. As expected, transport mediated by the mutant NaCTp.F500L was stimulated in accordance to previously published data^4^. This increase in transport was due to a lower K_m_ value resulting in a higher affinity of the mutated protein (Table 2) for citrate compared to the K_m_ value of the wild-type protein. For the wild-type NaCT protein-mediated citrate transport, a K_m_ value of 1 439 ± 270.8 µM was determined which is in the range of the previously published K_m_ value^18^. Furthermore, for the mutations NaCTp.G219R and −p.T227M, these transport data are also in accordance with published data^13^ using also HEK293 cells and with a published analysis investigating several NaCT mutations in COS-7 cells^14^. Interestingly, all mutations with a large effect on NaCT-mediated transport were classified as probably damaging in the PolyPhen2 Prediction (Prediction of functional effects of human nsSNPs – http://genetics.bwh.harvad.edu/pph2) and have high Grantham scores^17^ (Table 1).

**Figure 6:**
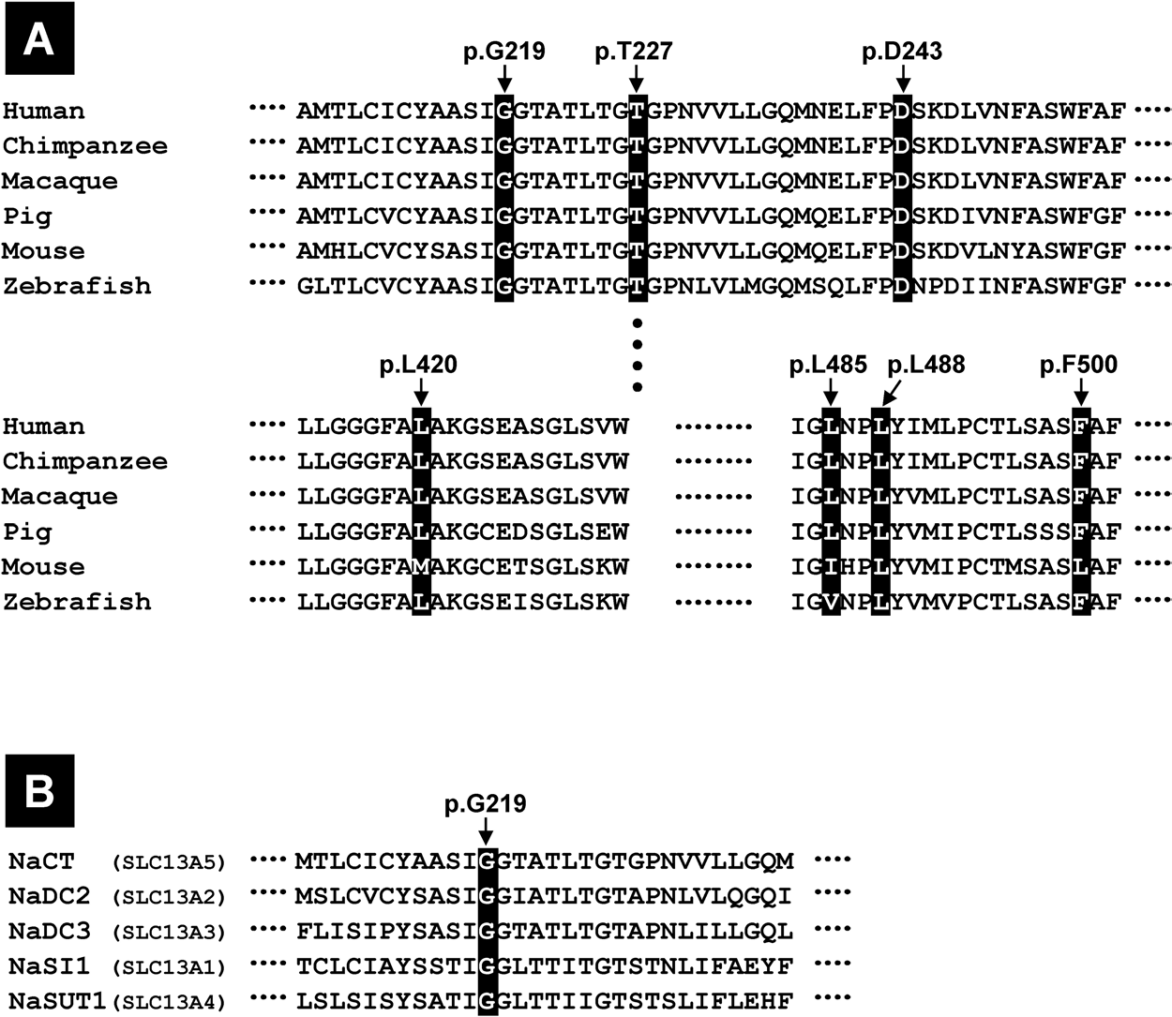
Clustal analysis of NaCT proteins from different species and of members of the SLC13 family. (**A**) For the NaCT clustal analysis of different species the sequences NM_001143838.2 (human), HM998308.1 (chimpanzee), HM998307.1 (macaque), KF728381.1 (pig), NM_001004148.4 (mouse) and HM998309.1 (zebra fish) were used. (**B**) For the NaCT clustal analysis of the human SLC13 family members the sequences NM_001143838.2 (NaCT), NM_003984.3 (NaDC2), NM_022829.5 (NaDC3), NM_022444.3 (NaSI1) and NM_012450.2 (NaSUT1) were used. Clustal analysis was performed using the HUSAR program package from the German Cancer Research Center^32^. All investigated mutations are indicated as boxes.

To rationalize the consequences of the mutations on a structural basis we established a computational model of the transport pore of the human NaCT protein and demonstrated that this model is in line with the experimentally observed transport data. Furthermore, this model is similar to the model of the *SLC13* gene family member NaDC3^19^ and highly similar [backbone RMSD (root-mean-square deviation) of 1 Å] to the NaCT model of the same working group found on their homepage (http://www.schlessingerlab.org/data/) but their model lacks the region around residue D243. Therefore, we generated a model including additional loop regions using a similar strategy. According to the dimeric oligomerization state of vcINDY, the homologous dicarboxylate transporter from *Vibrio cholerae*^16^ used as template, NaCT was also modeled as a dimer because inspection of the model suggests that dimerization is also sterically feasible for NaCT. Although a final determination of the dimerization state would require further experimental testing, none of the mutations investigated in the present study is close to the putative dimer interface and therefore the structural effects predicted for the mutations are not critically affected by the modeled oligomerization state. In the analysis of the vcINDY structure^16^, the authors detected one citrate molecule and one sodium molecule are bound per monomer and that conserved amino acids in the predicted transmembrane pore are important for ion binding and substrate translocation. In particular, the sodium ion binding site is highly conserved between human NaCT and vcINDY, which is also evident from a previous comparison of the human NaDC3 transporter and vcINDY^19^. This high conservation in the pore region between homologous residues is in line with the predicted functional consequences of the amino acid substitutions in the respective region of the NaCT transporter in this study. All amino acids investigated (except D243) are located within or near by the predicted transmembrane pore and are highly conserved among different species. A Clustal analysis demonstrated the G219, T227, D234 and L488 are found at identical positions in the alignment with NaCT proteins from human, mouse, pig, chimpanzee, macaque and zebrafish, whereas the amino acids L420 and F500 are conserved among most species with a variability only in mouse (Fig. 6A). Amino acid L485, where the mutation has a less distinct effect on citrate transport, is conserved in human, chimpanzee, macaque and pig.

L420 and L488 are located within predicted helices and replacement by helix breaking amino acids such as proline will disrupt this helical structure leading to a loss of transport activity and maybe affecting membrane insertion. Compared to the totally abolished transport of these two mutated proteins, the higher residual transport activity observed for the p.L485R mutation can most likely be attributed to the fact that the leucine sidechain is oriented away from the pore and can therefore be replaced by an arginine without causing an entire loss of structure. Amino acid residue F500 is also oriented away from the pore and the p.F500L mutation is predicted to enhance the flexibility of the NaCT structure due to poorer sidechain packing. This may be the explanation for the enhanced transport activity of this variant already published^4^ and shown here.

Interesting observations were made analyzing the effect of the two G219 mutants. In both mutants, NaCTp.G219R and −p.G219E, a complete loss of NaCT-mediated citrate transport activity could be observed. Molecular modelling reveals that the highly conserved glycine is located in sterically demanding position within a helix that tightly packs against A133 within a second helix (Fig. 5E,F). Due to the longer side chains of glutamate and arginine, they will form clashes with A133 thereby disrupting the protein structure, abolishing citrate transport and affecting plasma membrane insertion. The importance of this amino acid gets also obvious be analyzing both Clustal analysis (Fig. 6). G219 is not only highly conserved among NaCT proteins of different species (Fig. 6A) but is also conserved among all human *SLC13* gene family members (Fig. 6B). The role of all mutations for the membrane insertion of the NaCT protein has to be analyzed in detail.

Human NaCT is mainly expressed in liver and brain^20^ and the consequences of NaCT dysfunction have been studied in knock-out animals. These analyses showed that the absence of NaCT in mice promotes longevity and protects from insulin resistance and adiposity^10^. Furthermore, NaCT deficiency led to defective tooth and bone development characterized by the absence of mature enamel, decreased bone mineral density and impaired bone formation^11^. In contrast to the human “knock-out” phenotype, it has not been reported that knock-out animals show evidence of epileptic activity of other neurological signs, which may be due to species differences of transporter expression or other compensatory mechanisms. Neurons are incapable of synthesizing tricarboxylic acid metabolites and are therefore dependent on the uptake of these metabolites synthesized by astrocytes^21^. Therefore, insufficient transport of citrate and other TCA intermediates due to mutations in NaCT may result in brain energy failure causing epileptic encephalopathy. Interestingly, the same mutations may lead to differences in disease expression. This has been demonstrated in a recent publication by Anselm and coworkers were they investigated mutations in the *SLC13A5* gene identified in two siblings^15^. Both individuals carry the same mutations (p.G219R and p.Y82C) but only one of them had focal cortical dysplasia leading to brain surgery. These results suggest that additional factors downstream of the uptake transporter may be important for the development of this disease.

## Conclusion

The human transporter NaCT, encoded by the *SLC13A5* gene is important for teeth and bone development, energy metabolism, brain development and brain function by mediating the uptake of citrate and other intermediates of the tricarboxylic acid cycle from blood into cells (e.g. into hepatocytes and neurons). Mutations in the *SLC13A5* gene are associated with epileptic encephalopathy and developmental delay. We analyzed eight mutations in the *SLC13A5* gene leading to mutated NaCT proteins and could demonstrate that conserved amino acids within the predicted transmembrane pore are important for NaCT-mediated transport. The effect of all mutations on NaCT transport function could be explained on the structural basis of the NaCT transporter. Furthermore, some mutations may affect membrane insertion of the NaCT protein. Because HEK293 cells are not suitable in studying downstream effects of insufficient citrate uptake, additional studies using e.g. conditional knock-out mice models or *in vitro* studies with neural cells are required to analyze the effect of NaCT-deficiency in more detail and to explain obvious species differences.

## Methods

### Materials

[^14^C] Citrate (specific radioactivity 55 mCi/mmol) was purchased from American Radiolabeled Chemicals, Inc. (St. Louis, MO, USA). Unlabeled citrate was obtained from Fisher BioReagents, Fisher Scientific (Waltham, USA). Minimal essential medium, Dulbecco’s phosphate buffered saline, fetal bovine serum, G418-sulfate, penicillin streptomycin solution, Trypsin (0.05%)-EDTA (0.02%) solution, Lipofectamine 2000, and Optimem I were from Invitrogen/Thermo Fisher Scientific (Braunschweig, Germany). Sodium butyrate was purchased from Merck KgaA (Darmstadt, Germany). 12-Well tissue culture plates were obtained from Greiner Bio-One (Frickenhausen, Germany). Unless noted otherwise, all other chemicals were purchased from Carl Roth GmbH+ Co. KG (Karlsruhe, Germany).

### Cloning and Mutagenesis of the *SLC13A5* cDNA

The *SLC13A5* cDNA encoding the human NaCT (mINDY) transporter was cloned by a PCR-based strategy as described^18^. Mutations not described before were identified by database analysis using the following databases: the NCBI-SNP database (www.ncbi.nml.nhi.gov/snp); the central mutation and SNP database (www.hgvs.org/central-mutation-snp-databases) and the genetic variation database (www.humgen.nl/snp_databases.html). Each of the selected mutations in the *SLC13A5* gene was introduced separately into the expression vector pNaCT.31. Mutagenesis was performed using the QuikChange Lightning Multi Site-Directed Mutagenesis Kit (Agilent Technologies, Santa Clara, USA) according to the supplier’s instructions. Following primers were used for the mutagenesis reactions: oNaCTmutG219E: 5′-GGCCAGCATCGAGGGCACCGCCA-3′ (resulting in the plasmid pNaCT-mutG219E); oNaCTmutG219R: 5′-CGGCCAGCATCAGGGGCACCGCC-3′ (resulting in the plasmid pNaCT-mutG219R); oNaCT-mutT227M: 5′-CCACCCTGACCGGGATGGGACCCAA-3′ (resulting in the plasmid pNaCT-mutT227M); oNaCTmutD243N: 5′-CCAGATGAACGAGTTGTTTCCTCCCAGCAAGGACC-3′ (resulting in the plasmid pNaCT-mutD243N); oNaCTmutL420P: GGGCGGATTTGCTCCGGCTAAAGGATCCG-3′ (resulting in the plasmid pNaCT-mutL420P); oNaCTmutL485R: 5′-CGCTCCATCGGCCGCAATCCGCTGTAC-3′ (resulting in the plasmid pNaCT-mutL485R); oNaCTmutL488P: 5-CGGCCTCAATCCGCCGTACATCATGCTGC-3′ (resulting in the plasmid pNaCT-mutL488P); and oNaCTmutF500L: 5′-ACCCTGAGTGCCTCCCTTGCCTTCATGTTGC-3′ resulting in the plasmid pNaCT-mutF500L). Success of each mutagenesis was proofed by sequencing (LGC Genomics GmbH, Berlin, Germany).

### Transient transfection

For studying the functional consequences of the mutations, HEK293 cells were transiently transfected with the respective wild-type plasmid pcNaCT.31, the empty expression vector pcDNA3.1(+) as control and the plasmids pNaCTmutG219R, pNaCTmutG219E, pNaCTmutT227M, pNaCTmutD243N, pNaCTmutL420P, pNaCTmutL485R, pNaCTmutL488P and pNaCTmutF500L encoding the different mutated NaCT protein. Transient transfection has been performed as described^22^.

### Quantitative RT-PCR

Parental HEK293 cells were seeded at a cell density of 3 × 10^5^ cells per well in poly-D-lysine (0.1 mg/ml) covered 12-well tissue plates. Twenty-four hours after seeding, cells were transiently transfected with the respective expression plasmid. Each transfection was performed with 1.5 µg DNA per well using Optimem I and Lipofectamine 2000 (Invitrogen/Thermo-Fisher Scientific) according to the supplier’s instructions. After a further incubation of twenty-four hours, medium containing Lipofectamine 2000 and Optimem I was changed for standard cell culture medium and cells were further incubated for additional 24 hours. Messenger RNA was isolated by using RNeasy Mini Kit (Qiagen, Düsseldorf, Germany), according to the manufacturer’s instructions. Subsequently, RNA was reverse-transcribed into sscDNA, using Transcriptor Universal cDNA Master (Roche Diagnostics, Mannheim, Germany). LightCycler-based quantitative RT-PCR (Roche Diagnostics, Mannheim, Germany) was performed with following primers: Forward primer oNaCT-RT.for (5′-CCGCCTCAATCCGCTGTACA-3′) and oNaCT-RT.rev (5′-AGTCCTGAGGAGGGTAAGGG-3′) as described^23^. The *SLC13A5* mRNA expression in wild-type NaCT-transfected HEK293 cells, vector control-transfected HEK293 cells and in the HEK293 cells transfected with the different mutated cDNAs was calculated in relation to the expression of the housekeeping gene *ß-actin* and shown as relative *SLC13A5* mRNA expression in percent ± SD (with n = 3 independent expression analysis followed by one-way ANOVA with Bonferroni’s multiple comparison test).

### Uptake Assays

For uptake experiments, parental HEK293 cells were cultured and transiently transfected as described under quantitative RT-PCR. The uptake experiments using radiolabeled [^14^C]citrate were performed as described^24^. In brief, cells were washed with prewarmed (37 °C) uptake buffer (142 mM NaCl, 5 mM KCl, 1 mM K_2_HPO_4_, 1.2 mM MgSO_4_, 1.5 mM CaCl_2_, 5 mM glucose and 12.5 mM HEPES, pH 7.3). Subsequently, cells were incubated with 1 µM citrate in uptake buffer for 10 min at 37 °C. After incubation with the transport substrate, cells were washed three times with ice-cold uptake buffer, the cells were lysed with 0.2% SDS and the intracellular radioactivity was measured using a liquid scintillation counter (TriCarb 2800, Perkin Elmer Life Science GmbH, Rodgau-Jügesheim, Germany). Kinetic parameters (K_m_ and V_m__ax_ values) of wild-type NaCT and of the mutants NaCT −p.L485R and p.F500L (showing altered citrate transport) were determined with the same experimental setup using 1 µM, 100 µM, 1000 µM and 500 µM citrate. All uptake experiments were performed on two separate days in triplicates each (n = 6). In each well, parental HEK293 cells were separately transfected and subsequently used for the uptake experiments.

### Confocal laser scanning immunofluorescence analysis

Immunofluorescence was performed as described previously^22^. In brief, parental HEK293 cells were seeded at a cell density of 3.5 × 10^5^ cells per well in poly-D-lysine (0.1 mg/ml) covered 12-well tissue plates. Each well contained an autoclaved cover slip for higher adherence of the cells. After 24 hours, cells were transiently transfected with 1.5 µg DNA per well of the respective expression plasmids using Lipofectamine 2000 and Optimem I, according to the manufacturer’s instructions. Forty-eight hours after seeding, medium was changed and 24 hours later, cells were washed with Tris-buffered saline (TBS). After fixation in ice-cold 70% methanol, the cells were incubated with TBS/Triton (0.4%) for 10 min. Subsequently, the cells were incubated with 2% BSA/H_2_O for 45 min and then 90 min with a dilution (1:350 in 2% BSA/H_2_O) of monoclonal mouse anti-human-SLC13A5 antibody (Sigma-Aldrich Chemie GmbH, Taufkirchen, Germany) at room temperature. Afterwards, the cells were washed with TBS-Triton (0.1%) and TBS – Tween (0.05%), followed by incubation in a dilution (1:1 000 in 2% BSA/H_2_O) of the secondary antibody Cy2-coupled goat anti-mouse IgG (Dianova, Hamburg, Germany,) for 30 min at 37 °C. Subsequently, the cells were washed with TBS – Tween (0.05%), TBS Triton (0.1%), TBS and H_2_O. Then, the cover slips were fixated on microscope slides with mounting medium (Thermo Scientific, Dreieich, Germany). The immunofluorescence was detected with confocal laser scanning microscopy (Axiovert 100 M, Carl Zeiss GmbH, Jena, Germany). The images were sighted with the Zeiss LSM Image Browser version 4.2.0.121.

### Plasma membrane protein expression

The influence of the respective mutant on protein expression and localization was investigated using an immunoblot analysis after isolation of the total membrane protein fraction and of the plasma membrane fraction. For isolation of the total membrane fraction, 5 × 10^6^ parental HEK293 cells were seeded in 75 cm^2^ cell culture flasks for 24 hours under normal culture conditions. After these 24 hours, cells were transiently transfected with the respective wild-type or mutant expression plasmid or with the empty expression vector (32 µg plasmid/flask) as described above, grown for additional 24 hours before the HEK293 cell medium was changed. After additional 24 hours, cells were harvested and the plasma membrane fraction was prepared using the ProteoExtract Transmembrane Protein extraction Kit (Novagene, Merck-Company, Darmstadt, Germany) according to the manufacturer’s instructions. In brief, cells were washed two times with PBS at 4 °C and detached using a cell scraper. Then cells were transferred into a 15 mL falcon tube and centrifuged for 5 min at 1 000 g and 4 °C. The cell pellet was resuspended in 1 mL extraction buffer 1 supplemented with 5 µL protease inhibitor cocktail (supplied with the kit) and incubated for 10 min at 4 °C with gentle shaking. After centrifugation (5 min at 1000 g and 4 °C) the pellet (containing the membrane fraction) was resuspended in 200 µL extraction buffer 2B, supplied with 5 µL protease cocktail and incubated for 45 min at room temperature. After centrifugation (15 min at 16 000 g and 4 °C) the supernatant, containing the total membrane protein fraction was transferred to a fresh tube and the protein amount was calculated using a bicinchoninic acid assay (BCA Protein Assay Kit, Thermo Fisher Scientific, Rockford, Illinois, USA). For the isolation of the plasma membrane fraction the minute plasma membrane protein isolation kit (MoBi Tec, Goettingen, Germany) was used according to the manufacturer’s instructions. In brief, transiently-transfected cells were detached using a cell scraper, pelleted and resuspended in 500 µL buffer A. After incubation on ice, cellsuspension was vortexed and applied onto the filter unit. After centrifugation (30 s with 14 000 rpm) the pellet was vortexed again, centrifugalized, the supernatant was transferred into a fresh tube and centrifugalized again for 30 min with 16 000 rpm obtaining the total membrane fraction. This fraction was resuspended in 200 µL buffer B and intracellular vesicles were separated by centrifugation (5 min with 10 000 rpm). The supernatant was transferred into a fresh tube and the plasma membrane fraction was obtained by adding 1.6 mL ice-cold PBS and centrifuged again for 30 min with 16 000 rpm. The pellet containing the plasma membrane fraction was resuspended in PBS (with 0.5% Triton-100) and used for the immunoblot analyses.

Laemmli buffer (5×) and 20 µg of the respective protein (total membrane fraction or plasma membrane fraction) were mixed and denaturated at 95 °C for 5 min and then separated on 10% SDS-polyacrylamid gel. Subsequently, the proteins were transferred to Protran Nitrocellulose Membrane (Schleicher & Schuell, BioScience GmbH, Whatman Group, Dassel, Germany) and incubated with the primary monoclonal mouse anti-human-SLC13A5 antibody (Sigma-Aldrich Chemie GmbH, Taufkirchen, Germany) in a dilution of 1:500 for one hour at room temperature. After washing with PBS-Tween, the secondary goat-anti mouse antibody was incubated in a dilution of 1:2 000 for 30 min at room temperature. Imaging was performed with the Chemidoc XRS imaging system (Biorad, Munich, Germany) using ECL Western Blotting Detection Reagents (GE Healthcare). Membrane protein isolation was verified by stripping the membrane and restaining the immunoblot with an antibody directed against the membrane marker protein pan-cadherin (Sigma-Aldrich, Taufkirchen, Germany) under the same experimental conditions with a 1:250 dilution.

### Molecular Modelling

The structure of the human NaCT transporter was modeled using the structure of a homologous dimeric dicarboxylate transporter from *Vibrio cholerae* as a template [PDB code: 4F35^16^]. Both proteins exhibit a sequence similarity of 58%, which allowed to generate a NaCT model by homology modelling techniques. We noted that there already exists a model of NaCT (deposited on http://www.schlessingerlab.org/data/). However, this model lacks the region around residue D243 and therefore, we decided to generate a model including additional loop regions using a strategy similar to the one described^25^. Modelling was performed using Modeller9.10^26^ based on the INDY-NaCT sequence alignment published in^16^. The resulting model comprised residues F11-W116, R123-M159, K202-Y276, F314-G518, and K521-R549 of NaCT. In addition to the peptide chain itself, a sodium ion and a citrate molecule present in each subunit of the template crystal structure were also modeled in NaCT at the respective structure positions. The resulting model was verified using PROCHECK^27^ and WHATCHECK^28^ and revealed a good stereochemistry and no steric clashes. A comparison with the model by Schlessinger revealed a backbone RMSD (Root-mean-square-deviation) of 1.0 Å indicating that both models are highly similar. Mutations were modeled with SWISS-Model^29^ by selecting the lowest-energy sidechain rotamer for each mutated residue followed by 100 steps of energy minimization. Structure analysis and visualization was performed with visual molecular dynamics [VMD^30^] or RasMol (www.rasmol.org).

### Data analysis

The topology of the human NaCT transporter was predicted using the program TopPred1.10^31^. Net uptake values of citrate uptake were calculated by subtracting the uptake into vector-transfected cells from the uptake into NaCT-transfected (wild-type or mutated NaCT protein) cells. The citrate uptake was normalized to the protein concentrations of the cell lysate. Finally, all net uptake values of the mutant NaCT proteins are given in percent of citrate uptake into wild-type NaCT-transfected cells. Changes in transport rates were analyzed using one-way ANOVA with Bonferroni´s multiple comparison test. Affinity constants (K_m_ values) and maximal transport velocity (V_max_ values) were calculated using Michaelis-Menten kinetics. A p-value of ≤0.05 was required for statistical significance. All calculations were performed using GraphPad Prim 5.01.The Clustal analyses were performed using the HUSAR program package^32^ from the German Cancer Research Center, Heidelberg.

### Data availability

The datasets generated during and/or analysed during the current study are available from the corresponding author on reasonable request.

## Electronic supplementary material

Supplementary Data
