# Supplementary material for: Analysis of naturally occurring mutations in the human uptake transporter NaCT important for bone and brain development and energy metabolism
Source: Sci Rep. 2018 Jul 27;8:11330. doi: 10.1038/s41598-018-29547-8 (PMC6063891; doi:10.1038/s41598-018-29547-8)
Supplement: Supplementary file 1 — Supplementary Data [file 41598_2018_29547_MOESM1_ESM.pdf]

Analysis of naturally occurring mutations in the human uptake transporter NaCT important for bone and brain development and energy metabolism

Stefan Selch, Anja Chafai, Heinrich Sticht, Andreas L. Birkenfeld[3], Martin F. Fromm and Jörg König

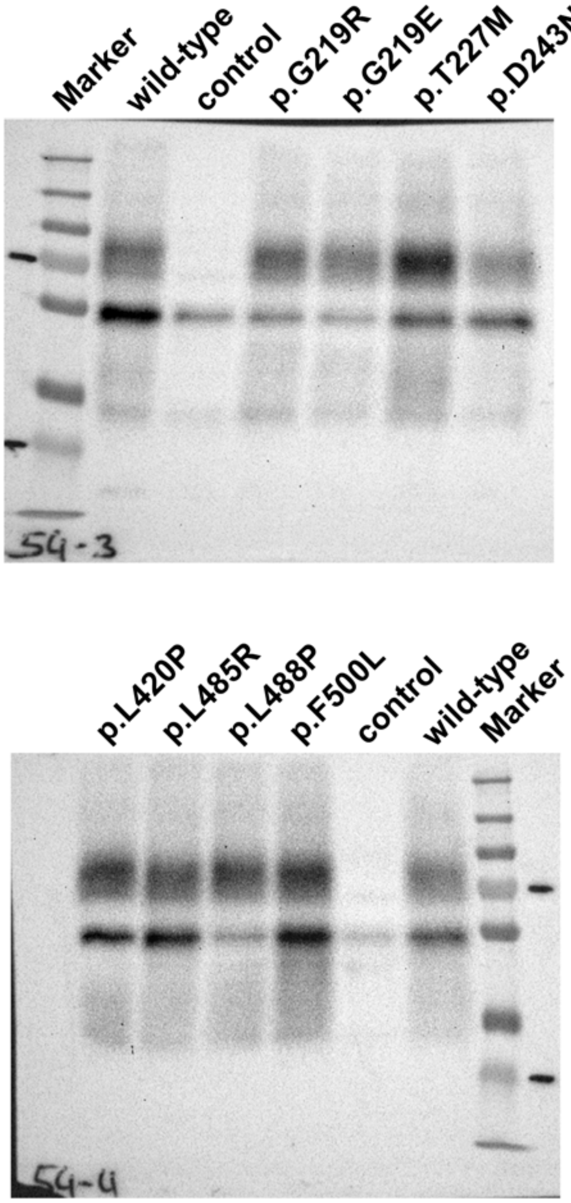

Supplementary Figure 1

Original immunoblot analyses of isolated total membrane fractions of transiently-transfected HEK293 cells expressing different variants of the NaCT protein. The cropped versions of these blots are presented in Fig. 2B.

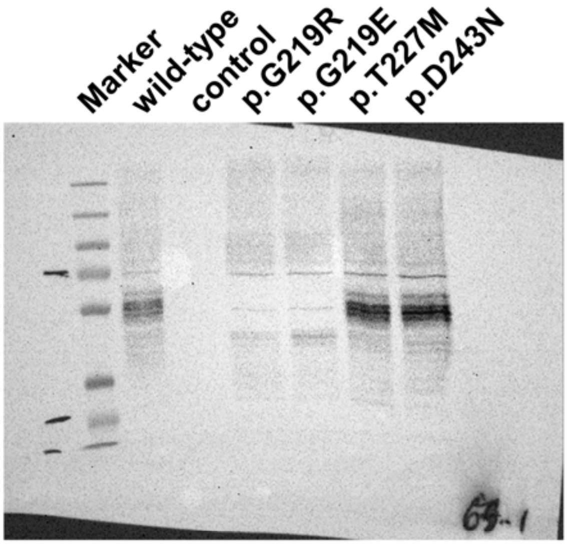

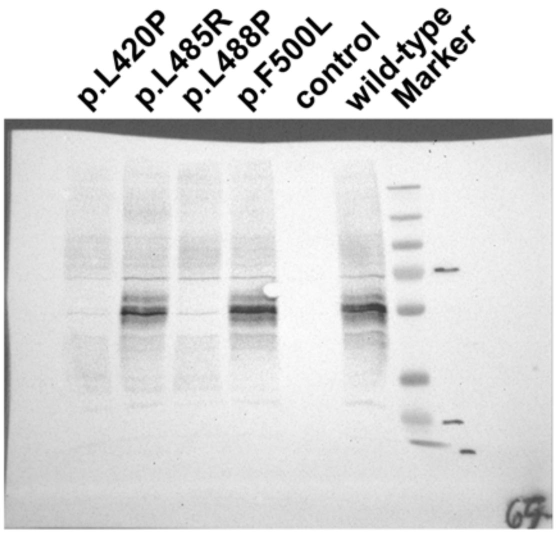

Supplementary Figure 2

Original immunoblot analyses of isolated plasma membrane fractions of transiently-transfected HEK293 cells expressing different variants of the NaCT protein. The cropped versions of these blots are presented in Fig. 2C.
